# Using Google Scholar institutional level data to evaluate the quality of university research

**DOI:** 10.1007/s11192-017-2532-6

**Published:** 2017-10-03

**Authors:** John Mingers, Jesse R. O’Hanley, Musbaudeen Okunola

**Affiliations:** Kent Business School, University of Kent, Canterbury, CT2 7FS UK

**Keywords:** Scientometrics, Research evaluation, Research excellence framework (REF), Google Scholar

## Abstract

In recent years, the extent of formal research evaluation, at all levels from the individual to the multiversity has increased dramatically. At the institutional level, there are world university rankings based on an ad hoc combination of different indicators. There are also national exercises, such as those in the UK and Australia that evaluate research outputs and environment through peer review panels. These are extremely costly and time consuming. This paper evaluates the possibility of using Google Scholar (GS) institutional level data to evaluate university research in a relatively automatic way. Several citation-based metrics are collected from GS for all 130 UK universities. These are used to evaluate performance and produce university rankings which are then compared with various rankings based on the 2014 UK Research Excellence Framework (REF). The rankings are shown to be credible and to avoid some of the obvious problems of the REF ranking, as well as being highly efficient and cost effective. We also investigate the possibility of normalizing the results for the university subject mix since science subjects generally produce significantly more citations than social science or humanities.

## Introduction

In recent years, there has been a major impetus to measure the quality of research at all levels, from the individual paper right up to the university and multiversity (Daraio et al. 2015; Millot 2015; Mingers and Leydesdorff 2015). Looking particularly at the university level, there are several approaches. First, there are global lists that rank the major universities throughout the world, for example the Times Higher World University Rankings (https://www.timeshighereducation.com/world-university-rankings), the Shanghai Academic Ranking of World Universities (http://www.shanghairanking.com), and the Quacquarelli Symonds World University Ranking (https://www.topuniversities.com/qs-world-university-rankings). These are all similar in collecting data on a range of factors such as teaching, research, and status or reputation and then combining them, often in a subjective way, to arrive at an overall quality metric (Hazelkorn 2015; Holmes 2013).

Second, there are single country evaluations, usually by peer review. The most well-known and elaborate is the UK’s research assessment exercise, currently called the Research Excellence Framework (REF) (http://www.ref.ac.uk) (Broadbent 2010; Moed 2008), although there are similar exercises in Australia—Excellence in Research for Australia (http://www.arc.gov.au/excellence-research-australia), New Zealand—Performance Based Research Fund (https://moetec.cwp.govt.nz/assets/Forms-templates-and-guides/PBRF-staff-guide.pdf), and Italy (Rebora and Turri 2013) for example. These generally involve university departments submitting samples of their research outputs, which are then evaluated by peer review panels. The process is time-consuming and extremely costly. The 2014 REF, for example, is believed to have cost over £250 m (Else 2015).

All of these methods rely, to a greater or lesser degree, on subjective judgement, whether it is the peer review of individual papers, judging the status of universities or how to combine different measures together. Also, they are all to some extent opaque in their methodologies—for example the REF does not give out grades by individual paper or person^1^ and the worldwide exercises do not reveal their background data. Bibliometric data, such as citations, is only used to a limited extent (Glänzel and Debackere 2009).

In this paper, we explore the extent to which readily available and free bibliometric data can be used to generate a credible evaluation of university research in a way that is transparent, non-subjective, and reasonably speedy. One could argue that the whole idea of producing such rankings should be avoided (Adler and Harzing 2009), but our view is that they do exist and are unlikely to go away, so we should try and ensure that they are as fair, transparent, and harmless as possible.

The data we will use is the institutional level data on citations that is available, although not widely known about, from Google Scholar (GS). Most academics will be aware that they may have a profile in GS which documents their institutional affiliation, their papers, and the citations they have received—if you type your own name into the GS search box you will see if you have a user profile. But it is also possible to search for an institutional domain name, e.g., “kent.ac.uk”, and then GS will produce data on all the researchers that are registered to that domain. This, in effect, produces citation data for the whole institution, which can be used to compare institutions in aggregate.

We collected this data for 130 UK university level institutions, all of which were included in the 2014 REF. We explore various metrics based on this data and compare it with three metrics available from the REF—GPA, power, and intensity. We compare both in absolute terms and in terms of rankings. We then consider the issue of normalization. It is well-known that the sciences, especially medicine and biology, cite much more frequently than the social science or humanities (Bornmann and Marx 2015; Waltman and van Eck 2013). To account for this, citation data should be normalized in some way. For a paper, this would be relative to the level of references or citations in a given field. At the university level, the problem is that universities have different mixes of subjects, some being almost exclusively science-based, others having virtually no science at all. The latter, therefore, would be disadvantaged in terms of absolute citation numbers. We explore a method of correcting the data for this effect.

## Methodology and data collection

### Methodology

There are two primary sources of citation data—Web of Science (WoS) and Scopus, which collect and validate data from a subset of journals, and Google Scholar, which searches the web looking for citations to specific papers and books. The strengths and weaknesses of the two sources have been well documented (Adriaanse and Rensleigh 2013; Bornmann et al. 2016; Delgado-López-Cózar and Cabezas-Clavijo 2012; Harzing and Alakangas 2016; Mingers and Lipitakis 2010; Prins et al. 2016). Broadly speaking, WoS and Scopus produce high-quality and comprehensive data for the journals that they cover, but they do not generally include books and their coverage is incomplete, especially in the social sciences (around 50%) and arts and humanities (around 30%). GS has a very good coverage (up to 90%) and is roughly the same for all disciplines. On the other hand, its data can be unreliable; often generating multiple versions of the same paper, and it sometimes includes non-research outputs such as teaching notes and home pages.

The user interface to GS is simplistic and it offers few facilities, for example field lists of journals, but because of its coverage it is an important source of data for the social sciences and arts and humanities. It is also the case that there is little documentation available and, in fact, this paper utilizes a search facility that is little known because it is undocumented.^2^ With a web browser, one can type the following search into the address bar: https://scholar.google.com/citations?mauthors=xxxxxxx.xxx&hl=en&view_op=search_authors where xxxxxx.xxx is the domain name of the institution one is interested in. For example, “kent.ac.uk” would return the University of Kent in the UK.^3^

The result is a list of the academics affiliated with the institution (through their email domain name) that GS has recorded. The results appear in order of total number of GS citations for each academic, ten per page, but further pages can be searched until the list is exhausted. Moreover, if one clicks on an individual, you will access his/her GS profile, which includes a list of papers and further citation statistics, including *h*-index (Bornmann et al. 2008; Egghe 2010; Franceschini and Maisano 2010; Xu et al. 2015) and i10-index (a GS-specific metric that is the number of papers with at least 10 citations), together with alternative versions of each for the past 5 years.

We should at this point consider the accuracy of the Google Scholar data. Numerous studies have shown that there is a significant degree of error in the data although this is counterbalanced by the greater coverage in terms of both type of output and discipline (Delgado-López-Cózar and Cabezas-Clavijo 2012; García-Pérez 2010; Harzing 2013, 2014; Martín-Martín et al. 2014; Prins et al. 2016). The main studies have concerned errors in the citations themselves—multiple records for essentially the same paper and citations from a range of non-research sources. This is of some concern but in this study we are working at a high level of aggregation—whole institutions—and there is no reason to suspect that it will affect particular institutions differentially. We are concerned with the relative number of citations, not the absolute number.

Of more concern is a different source of error—the accuracy of the list of academics in the institutional profile. This depends on scholars actually having a Google profile, which in turn is related to their email address. There can be errors of two kinds—commission and omission. The former is when an academic appears in the list but should not, perhaps because they have left the institution; the latter is when they do not appear in the list but should, perhaps because they do not have a Google profile. It is not possible to evaluate the level of error in general because we cannot access institutions’ HR databases but we can do so for our own institution—the University of Kent.

In terms of commission errors, two staff had retired but were Emeritus Professors, two had left but still had honorary status and thus valid emails, and one had indeed left but a search showed no subsequent post to replace Kent. In terms of omission errors, this is harder to spot as you need to find researchers at an institution who were not in the list but might have high citations. We used *Scopus SciVal* to identify researchers at Kent together with their citations (which were only between 2014 and 2016) which gave us a list of highly cited staff. We then looked each one up Google Scholar to estimate if they should have been included in the list (this was only an estimate because the error checking was carried out some time after the original data collection). We identified two researchers who we thought should have been included.

Overall, the error rate on this admittedly small sample seems to be only around 5%. We feel that this is not a high error rate and there is no reason to suppose that there will be systematic differences between universities. In time, as Google Scholar becomes used more extensively for research evaluation, we would expect universities to monitor this and ensure both that their staff have profiles, and that out of date profiles are removed thus improving the quality of data.

The idea of this paper is to calculate a central tendency of these metrics, e.g., the mean or median, as a measure of the research impact of the institution as a whole and then compare the resulting rankings with rankings produced by the REF. There are several decisions in the data collection that one must consider:

- How many academics should be used from each institution? Taking a small sample of universities showed that the number of academics varied widely, from many hundreds for a large research intensive institution, to only 20 or 30 for a small teaching institution. There is, in fact, a test version of a worldwide university ranking based on GS data (http://www.webometrics.info/en/node/169). This, however, uses only the first page of data, i.e., the top 10 academics, while ignoring the first academic for reasons of “representativeness”. It then uses the total of the remaining academics’ GS citations. Our view was that ten academics were insufficient to give proper representation, especially for universities that did not have much science since it could be dominated by a small group in a field of very high citations.
- On the other hand, going into the hundreds would not work for those institutions with only a small number of academics. As a compromise, we used the top 50 academics.
- What should be done with institutions having less than 50 academics? The issue here is whether to examine less than 50, or to add zero entries to reach 50. Given that we will be using the central tendency, we opted for the second option, which is also what the REF did when less than the required minimum of four papers was submitted.
- Which primary metric should be used? There are six choices—citations, i10-index and *h*-index, either total or over the previous 5 years. We rejected the i10 indicator as it is not well researched and only by GS. We collected data for the others and the final choice is discussed in the results section.
- Which measure of central tendency should be used—mean or median? Again, we investigated both and discuss this in the results.

### Data collection

We began the data collection manually, but it turned out to be a significant task, as there were approximately 130 universities and each one required 50 × 6 data items to be recorded. It was subsequently decided to automate the process by coding a bespoke program in R to scrape the data from GS. This turned out to be far quicker once the program was written and tested.

The REF data was obtained from the HEFCE data site (http://www.ref.ac.uk) and from the Times Higher (https://www.timeshighereducation.com/news/ref-2014-results-table-of-excellence/2017590.article), which produced the ranking tables.

In terms of the REF data, there are three different metrics available. The major one, which is used to create the main ranking table, is known as the grade point average (GPA). The 2014 REF methodology was complex. Institutions submitted selected research active staff (the number decided by each institution) and for each staff member up to four research outputs were provided. They also submitted a research environment statement and a set of impact case studies that depended on the number of staff submitted. To give an idea of the size of the 2014 REF, there were 1911 submissions (departments, school, research centers, etc.), 50,000 staff, 191,000 research outputs (articles, monographs, books, book chapters, etc.), and 7000 impact case studies. Each research output was read and graded on a 5-point scale (0*–4*), where 0* indicated no research content and 4* indicated “world leading” quality. The case studies and the environment statements were graded on the same scale. A profile was then created for each unit showing the proportion of 1*–4* outputs in each of the three categories. These were combined into a weighted average to arrive an overall profile for each unit. Unit-level profiles were independent of the number of staff submitted. The Times Higher subsequently calculated a mean value across units to give a final GPA for each institution.

Profiles were highly sensitive to the proportion of staff submitted. If a unit or institution submitted only its very best researchers with top quality research outputs, it could easily increase its GPA. Conversely, if a unit submitted a large proportion of its staff, it usually resulted in a lower GPA. A considerable number of departments and universities played this game very seriously, which our results clearly show. Because of the weakness with GPA, two other measures were calculated and used for rankings. One, called “power”, is the GPA multiplied by the actual number of staff submitted. This serves as a measure of the research contribution of a given unit/institution. The other, called “intensity”, is the GPA multiplied by the percentage of eligible staff submitted. Units/institutions with full submissions, i.e., non-selective, did well on intensity and power if they were large, but usually poorly on GPA. Those that were highly selective did well on GPA but poorly on power and intensity. Clearly, this is not an ideal situation, given that there was confusion about which ranking to use and institutions could cherry-pick which one was best for them (Mingers and White 2015).

## Results

### Exploration of the data

We have two main types of data, that from the REF which has three variables—GPA, intensity, and power—and that from GS citations—mean and median total citations, mean and median 5-year citations, and mean and median 5-year *h*-index. Further explanation and summary statistics are shown in Table 1.

**Table 1:** Summary statistics

Indicator	Description	Mean	Median	SD	Coeff of var	Skewness
GPA	REF quality score (1*–4*)	2.72	2.75	0.42	15.4	− 0.67
Power	GPA × no. of staff submitted	1211	631	1538	127	2.45
Intensity	GPA × % staff submitted	1.43	1.09	0.94	65.4	0.28
Mean cites	Mean total citations for top 50 researchers	7062	3110	9719	137.6	2.51
Median cites	Median of above	4956	1899	7450	150.3	2.75
Mean 5-year cites	Mean citations for top 50 researchers over last 5 years	3595	1716	4738	131.8	2.41
Median 5-year cites	Median of above	2534	1085	3664	144.6	2.52
Mean 5-year *h*-index	Mean *h*-index of top 50 researchers over last 5 years	21.3	18.4	14.7	69.3	0.78
Median 5-year *h*-index	Median as above	19.23	16.8	14.3	74.6	0.78

It is noticeable that the GS citation variables are all highly skewed even though the values are the means for 50 academics. Median citations are significantly lower than the means.

**Fig. 1:**
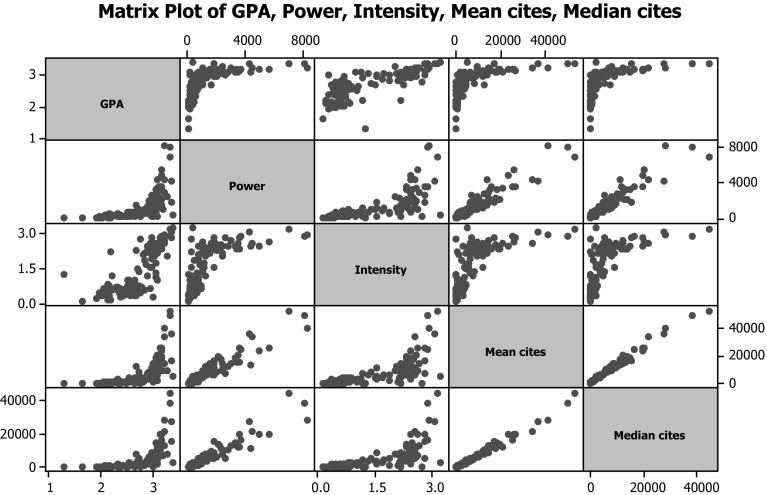
Matrix plot of scattergrams for selected indicators

Table 2 shows the Pearson correlation coefficients and Fig. 1 the matrix of scatter plots for each research quality indicator. For reasons of clarity, we only show in Fig. 1 one of the GS citation measures (mean citations) together with the REF metrics. One obvious feature of many of the plots is their non-linearity since citation data is generally highly skewed. The exception is Power which is itself exponential resulting in an overall linear relationship to citations. This will be examined further later.

**Table 2:** Correlation matrix

GPA	Power	Intensity	Mean cites	Median cites	Mean 5-year cites	Median 5-year cites	Mean 5-year *h*-index	
Power	0.625							
Intensity	0.772	0.700						
Mean cites	0.642	0.945	0.711					
Mediancites	0.628	0.945	0.698	0.992				
Mean 5-year cites	0.650	0.935	0.717	0.992	0.986			
Median 5-year cites	0.638	0.937	0.706	0.989	0.994	0.989		
Mean 5-year *h*-index	0.820	0.887	0.812	0.916	0.901	0.923	0.910	
Median 5-year *h*-index	0.820	0.891	0.808	0.913	0.900	0.916	0.909	0.996

Looking at the correlations, we can see the following main features:

1. The correlations among GS citation measures are all very high (the lowest being 0.90 between median citations and mean 5-year *h*-index).
2. Correlations among the REF measures are less strong (0.625–0.772).
3. The Power measure shows a strong correlation with all GS citation metrics since all these metrics factor in the number of high-quality research outputs.
4. Intensity shows a stronger correlation with the citation metrics than it does with GPA indicating that this metric better accounts for quantity than pure GPA.
5. The* h*-indices are more highly correlated than the other citation metrics with the REF measures.
6. In spite of generally high correlations observed (0.625–0.996), the different metrics nonetheless yield very different rankings as is generally the case (Mingers and Yang 2017).

**Fig. 2:**
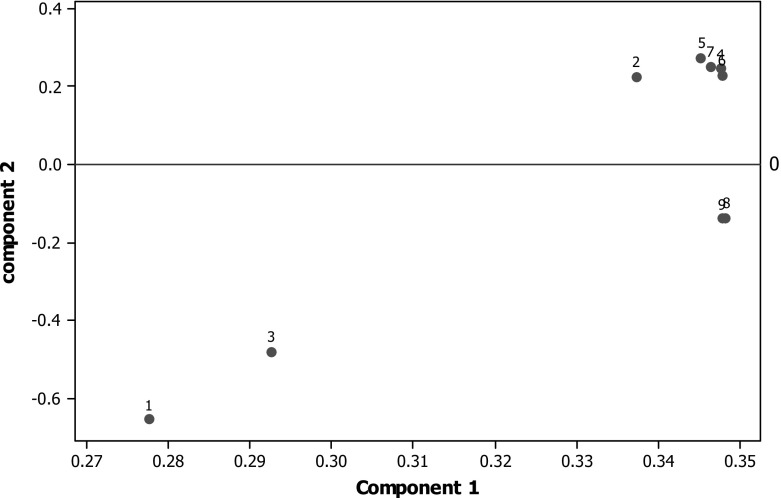
First two principal components for the raw indicators. *1* GPA, *2* power, *3* intensity, *4* mean cites, *5* median cites, *6* mean 5-year cites, *7* median 5-year cites, *8* mean 5-year *h*-index, *9* median 5-year *h*-index

Figure 2 shows a plot of the first two principal components for the raw metrics. All variables are positive on component 1, which can be interpreted as the overall research strength. On component 2, GPA and Intensity (and marginally the* h*-indices) stand opposed to the GS citation measures, so this component would appear to contrast total research contribution against a selective contribution. As can be seen, the citation measures group extremely close together along with Power; the h-indices form a separate group; GPA and Intensity another distinct group.

Overall, these results show that among the REF metrics, Power is distinct from the other two and very similar to the GS citation metrics.

**Fig. 3:**
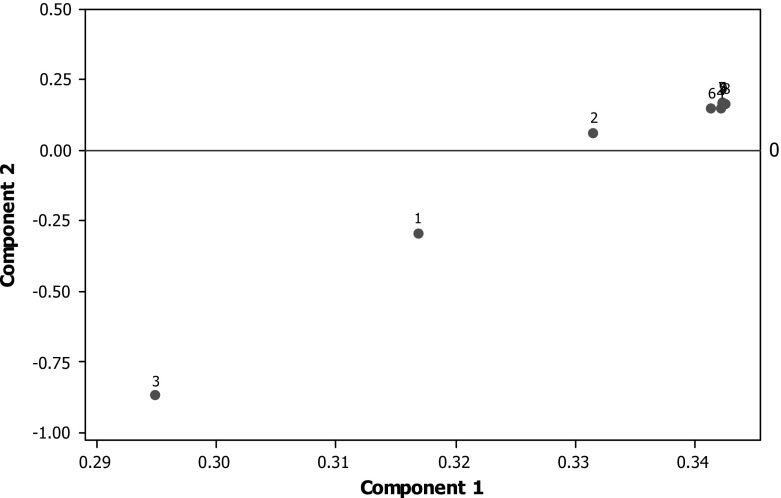
First two principal components on ranked indicators. *1* GPA, *2* power, *3* intensity, *4* = mean cites, *5*  median cites, *6* mean 5-year cites, *7*  median 5-year cites, *8* mean 5-year *h*-index, *9* median 5-year *h*-index

The next stage was to consider the rankings produced by these metrics, i.e., how institutions compare to each other for each metric in turn. This was done partly to overcome the effects of non-linearity and partly because ultimately it is the rankings which are important to an institution’s reputation rather than the absolute values. The principal component plot for institutional rankings is shown in Fig. 3. The main difference from Fig. 2 is that all the GS citation metrics are extremely close but the REF metrics are more spread apart. Power is still close to the citation metrics.

### Comparison of rankings

We have three REF indicators and six GS indicators so in order to make a sensible comparison we need to focus on selected ones. The REF indicators each measure different things—pure research quality independent of size (GPA), research quality relative to the proportion of research staff (intensity), and total research contribution (power)—while the GS metrics are essentially different measures of the same thing so it makes the most sense to reduce these. The *h*-index is a derived indicator and subject to various criticisms (Egghe 2010; Franceschini and Maisano 2010; Gingras 2014, 2016) so we chose to drop these. It also seems sensible to pay more attention to recent research (at least for the purposes of the REF) and so we chose to drop total citations, leaving the 5-year citations. Finally, because of the extreme skewness for 5-year citations, it was deemed better to use the median rather than the mean, thus leaving 5-year median citations as the primary GS indicator.

Table 3 shows the rankings for the top 25 UK universities according to the three research indicators (the full Table is available from the online version of the paper). Table 3 is ordered with respect to the GS ranking produced using the median 5-year citations. *Prima facie*, having Cambridge and Oxford as the top two universities seems reasonable, as almost all world-wide rankings have these as the top UK universities and it would seem rather strange for the REF rankings not to have them located there. Next come Imperial and UCL, which are two major science-based universities in the UK. The rest of the top 10 more or less follow the power ranking, which is what one would expect from the correlation results presented earlier. In 5th place is Southampton, another science-intensive university, which also 8th for intensity and 11th for power based on its large submission to the REF. Manchester, a very large university, was significantly lower on both GPA and intensity. This is slightly anomalous but suggests that its sheer size overcomes some weakness with regard to research quality and the percentage of staff submitted.

**Table 3:** Ranking of the top 30 universities based on median 5-year citations (including the Institute of Cancer Research which came 1st on GPA and intensity)

Institution	Staff submitted to REF	Median 5-year cites	GPA	Power	Intensity	Mean 5-year cites adjusted for subject mix
University of Cambridge	2088	1	5	3	2	3
University of Oxford	2409	2	4	2	5	2
Imperial College London	1257	3	2	8	3	57
University College London	2566	4	8	1	4	7
University of Southampton	1113	5	19	11	8	23
University of Manchester	1561	6	17	5	27	12
King’s College London	1369	7	7	6	17	11
University of Edinburgh	1753	8	13	4	12	6
University of Bristol	1138	9	12	9	6	19
University of Birmingham	1065	10	32	14	23	16
University of York	643	11	16	23	32	9
London School of Economics and Political Science	532	12	3	28	7	1
University of Exeter	736	13	30	21	19	4
University of Warwick	931	14	9	15	11	15
Queen Mary University of London	671	15	11	22	34	17
University of Glasgow	1099	16	25	12	15	21
University of Leeds	1149	17	21	10	36	22
University of Sheffield	1043	18	15	13	33	41
Durham University	740	19	20	20	25	8
Newcastle University	888	20	26	16	26	34
University of Nottingham	1404	21	29	7	28	29
University of Aberdeen	597	22	47	29	57	38
University of Sussex	501	23	40	34	42	5
University of St Andrews	519	24	22	32	16	20
Lancaster University	580	25	18	26	29	14
University of Reading	590	26	39	27	21	28
London School of Hygiene and Tropical Medicine, University of London	314	27	10	46	13	96
University of Bath	462	28	14	35	35	55
University of Dundee	396	29	38	39	49	42
Royal Holloway, University of London	378	30	27	40	31	13
Institute of Cancer Research	103	52	1	87	1	104
Mean absolute difference in ranks relative to median 5-year cites for all 130 universities		0	12.7	8.7	16.5	

The London School of Economics (LSE) is an interesting case which we will discuss later when we consider normalizing for disciplinary mix. This is a high-quality institution but has no science, and science subjects tend to generate higher levels of citations. It was 3rd for GPA and 7th for intensity but only 28th for power, reflecting its small size (only 532 staff submitted, one of the lowest in the top 30). In terms of GS citations, it came 12th, which is much higher compared to its power ranking. Looking further down the Table, we can find other instances where the power measure is affected by sheer size, for instance Nottingham which is 7th on power but in the 20’s for the other indicators.

There are some interesting cases that do not appear in Table 3 because they are not in the top 30 for GS citations. Perhaps the most glaring is Cardiff, which had a very selective submission (as the VC made clear in his statement about REF strategy). This led to them coming 6th out of all UK universities based on GPA. More realistically, it was 18th for power and only 50th for intensity. Cardiff came 34th for GS citations. Conversely, Brunel did very poorly on GPA, coming 75th because it had a full submission, in spite of being 40th for intensity and 33rd for both power and citations.

The position of very specialized institutions is also interesting. Although the main REF results exclude pure single-subject institutions such as art and drama schools or veterinary colleges, they do include, for example, the Institute of Cancer Research (ICR) and the London School of Hygiene and Tropical Medicine (LSHTM), both of which are very small and only entered in two REF units for assessment. ICR was actually top overall on the GPA measure, which does seem peculiar given its limited coverage of subjects. It also came top for intensity, although it only submitted 103 staff. In contrast, it came 87th for power and 52nd for GS citations.

The final row of the Table shows the mean absolute difference in ranks (among all 130 universities) between the REF and GS citation metrics. It further confirms that the GS citation metric is closest to power.

### Taking account of subject mix

As mentioned previously, it is a notable feature of citations that volumes differ significantly between different subjects with science, especially medicine and biology, producing very much greater levels of citations that social science and even more so arts and humanities. A separate but related problem is that citation sources such as Web of Science and Scopus have a greater coverage of the sciences, a problem that Google Scholar does not suffer from.

This means that in order to be able to compare papers, journal or people from different disciplines, the data has to be normalized to account for the disciplinary differences. When looking at an institutional level, normalization does not usually take place on the assumption, at least presumably, that any university will have a wide coverage. In truth, this is not the case. For instance, within the UK, Imperial College is almost entirely science with no arts or social science, while LSE is the opposite with only social science. It might, therefore, be expected that indicators based on citations will be biased in favor of the science-intensive universities and that the data ought to be corrected for this bias.

The first issue that arises is determining the extent of the mix of science and non-science. One could easily estimate this from the REF data. Overall, the REF was split into 36 field-based sub-panels that were amalgamated into four main panels—Panel A covered medicine, biology and agriculture, Panel B covered science and engineering, Panel C covered social science, and Panel D the arts and humanities. So, broadly speaking, Panels A and B were science and C and D were non-science. Since the REF data included the number of staff submitted by each university to each panel, we calculated the percentage of each submission that was classified as science.

We could then see what proportion of the GS citations of an institution could be explained by the percentage of science using linear regression. A high degree of non-linearity was observed between percent science (independent variable) and GS citations (dependent variable), owing to the large underlying skewness of citations. To correct for this, we took the natural logarithm of GS citations, i.e., log_e_(citations). This, however, gave rise to a separate problem. Specifically, based on median citations, a number of institutions had values of zero, which is undefined for natural log transform. Consequently, it was decided for the purpose of this exercise to use the *mean* 5-year citations rather than the median as the dependent variable.

**Fig. 4:**
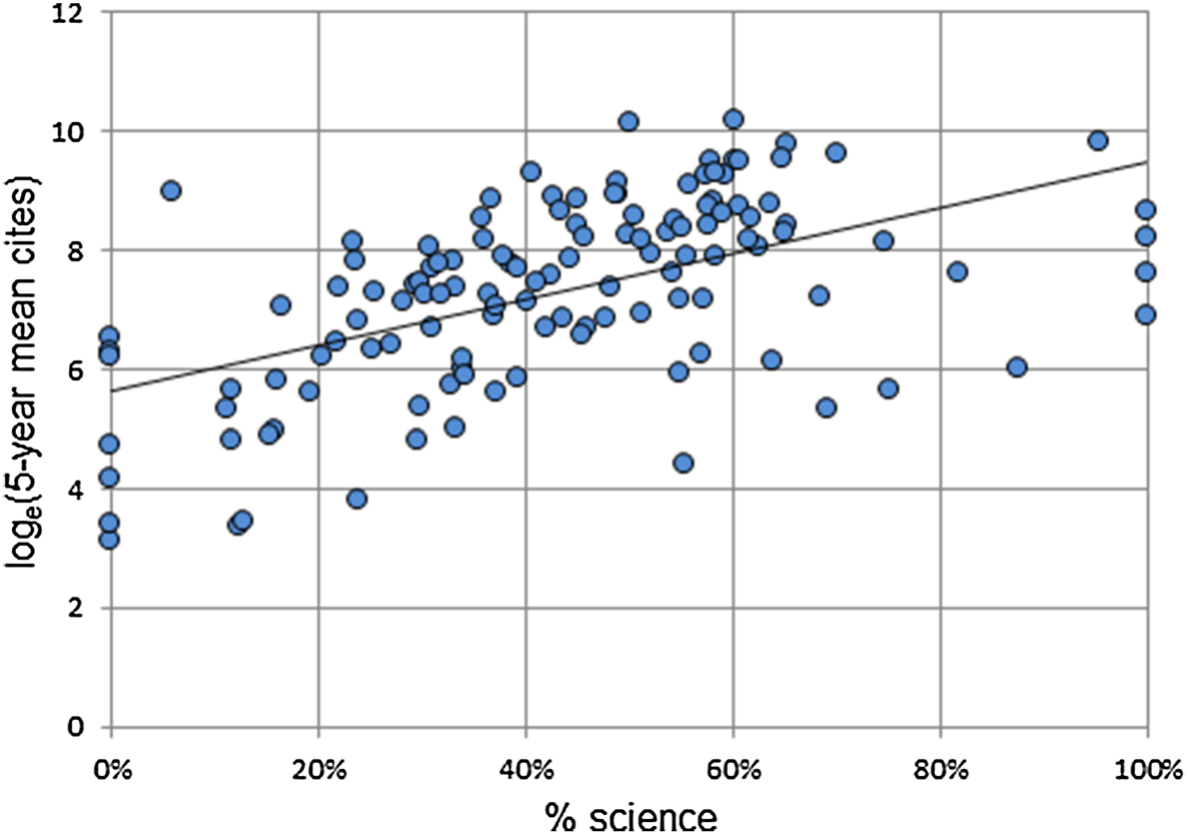
Regression of log_e_(mean 5-year cites) against % science mix

Regression results are reported in Fig. 4 and Table 4. The resulting regression with a single predictor variable (% science) was highly significant. The overall F-value for the regression with was 52.2 (*p* = 4.4×10^−11^), the adjusted *R* ^2^ was 0.29, and both the intercept and slope were significant at < 1% level.

**Table 4:** Regression of log_e_ (mean 5-year) cites against % science mix

Summary output	
*Regression statistics*	
Multiple *R*	0.542621
*R* ^2^	0.294437
Adjusted *R* ^2^	0.288793
Standard error	1.318602
Observations	127

ANOVA					
	*df*	SS	MS	*F stat*	*P* value
Regression	1	90.69732	90.69732	52.16354	4.4E−11
Residual	125	217.3389	1.738711		
Total	126	308.0362			

	Coefficients	Standard error	*t* stat	*P* value	Lower 95%	Upper 95%
Intercept	5.648028	0.257122	21.96636	9.68E−45	5.139152	6.156904
% Science and medicine	3.826368	0.529789	7.222433	4.4E−11	2.777849	4.874887

The resulting regression equation was: log_e_(mean 5 year cites) = 5.65 + 3.83 × % science.

**Fig. 5:**
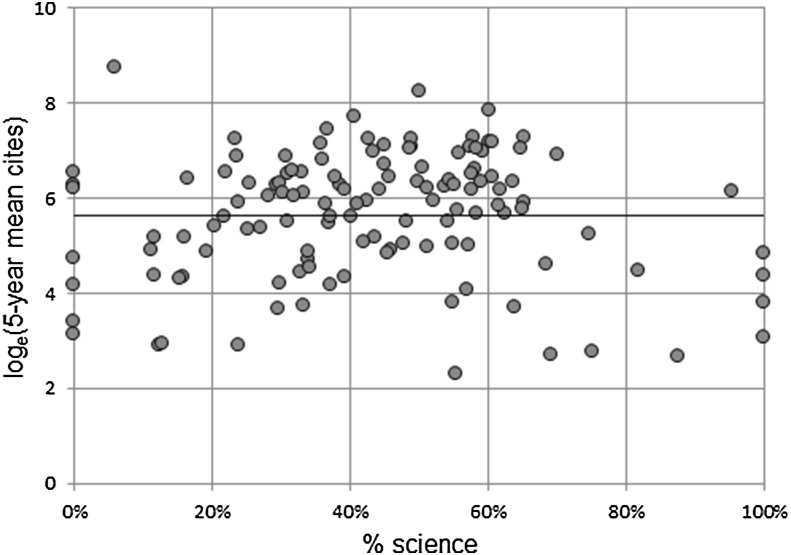
Graph of log_e_(mean 5-year cites) adjusted for % science mix

This means that a 1% increase in the percentage of science mix will produce an increase of e^3.83^ or 46 mean citations. This can be used to remove the effects of the subject mix from the raw figures. The results are shown in the fourth column of Table 5 and a plot of the adjusted data is shown in Fig. 5. Figure 5 clearly shows that the effect of subject mix has been removed. We ranked the universities on this adjusted indicator and the results are shown in final column of Table 3.

**Table 5:** Mean 5-year citations adjusted for subject mix for top 20 and bottom 20 institutions

Institution	% Science and medicine	Mean 5-year cites	Mean 5-year cites adjusted for science
London School of Hygiene and Tropical Medicine, University of London	100.00	5752.00	124.9
Institute of Cancer Research	100.00	3636.10	78.9
St George’s, University of London	100.00	2024.34	43.9
Liverpool School of Tropical Medicine	100.00	988.29	21.5
Imperial College London	95.39	17,902.48	463.7
Glyndwr University	87.61	409.92	14.3
Cranfield University	81.77	1968.24	85.9
Abertay University	75.20	284.50	16.0
Heriot-Watt University	74.73	3307.32	189.0
University of Southampton	70.09	14,727.42	1005.2
University of West London	69.19	206.00	14.6
University of Hertfordshire	68.51	1367.78	99.2
University College London	65.26	17,433.78	1431.8
University of Liverpool	65.17	4443.70	366.2
University of Surrey	64.96	3893.34	323.5
University of Bristol	64.88	13,764.12	1146.9
Edinburgh Napier University	63.90	459.06	39.7
University of Sheffield	63.64	6528.48	570.4
University of Strathclyde	62.44	3140.84	287.4
University of Bath	61.69	5057.52	476.3
*Bottom 20*			
Goldsmiths, University of London	22.06	1587.84	682.2
Leeds Beckett University	21.64	617.76	269.7
Edge Hill University	20.43	485.84	222.1
Canterbury Christ Church University	19.27	270.90	129.5
University of Roehampton	16.37	1146.44	612.4
Birmingham City University	16.00	325.46	176.4
Liverpool Hope University	15.83	141.73	77.3
York St John University	15.40	132.86	73.7
Newman University	12.88	30.86	18.8
University of Wales Trinity Saint David	12.37	28.66	17.8
University of Winchester	11.71	276.20	176.4
University of Chichester	11.61	122.86	78.8
University of Cumbria	11.19	204.18	133.0
London School of Economics and Political Science	5.83	7893.82	6313.5
SOAS, University of London	0.00	681.80	681.8
Bath Spa University	0.00	532.12	532.1
University of Gloucestershire	0.00	495.20	495.2
Falmouth University	0.00	22.46	22.5
St Mary’s University, Twickenham	0.00	29.56	29.6
Leeds Trinity University	0.00	112.50	112.5
Bishop Grosseteste University	0.00	63.62	63.6

As one might expect, this makes a major difference to the final ranking, especially for science-intensive universities, although it is noteworthy that Cambridge and Oxford still come out at on top because they both have a relatively balanced mix. The major “losers” of the adjusted GS citations ranking are Imperial College, Southampton, Aberdeen, Sheffield, and Newcastle. The biggest “winner” is LSE, which moves up to first place. One could certainly argue that is justified given its high number of citations in spite of having virtually no science at all. Other winners were Liverpool, Essex, Birkbeck, and Goldsmiths College.

Whilst we would not go so far as to suggest that this fairly blunt form of normalization should actually be used in practice, it does show the extent of the handicap non-science universities have when measured on raw citations. Having said that, the adjusted GS citations ranking is not only different from the original GS citations ranking (correlation 0.84) but is also different from the REF, e.g., the correlation with GPA is only 0.70, which is more similar to the GS citation ranking. What does seem clear from all this is that the REF peer review appears to favor science-intensive universities over others.

## Conclusions

In this paper, we explore the extent to which readily available citation data from Google Scholar can be used to form a credible evaluation of a university’s research and compare this with the UK Research Excellence Framework results.

We argue that the GS citations ranking is credible in that there are no glaring instances where a university is clearly misplaced. More than that, we suggest that in many ways the resultant ranking is actually superior to that produced by the REF, as well as hugely less costly and time-consuming.

The REF produced three distinct rankings, each of which has very definite biases that can and were exploitable by universities. GPA favored those who were very selective in their submissions, a strategy that has the serious potential to affect the careers of many staff. Moreover, it generated highly anomalous results in some cases, including Cardiff University, which came 6th overall. Intensity went in the opposite direction, favoring those institutions who submitted a high proportion of staff even when overall research quality was less strong. Finally, power favored those with a large submission, whether or not quality was high or very intensive. Examples of these are detailed in the paper.

In contrast, the ranking based on GS citations steers a middle path between GPA, intensity, and power. Although most similar to power, because it does take into account the absolute amount of research, it does not favor pure size to such an extent, as the examples of LSE and Nottingham show. In fact, it could be argued that all of the REF rankings, produced at such a cost, have significant flaws.

Additionally, using GS citations does not allow the kind of game playing that the REF does in terms of the proportion of staff submitted. No doubt citations can be massaged, but not in such a direct way that would lead to the anomalies observed with Cardiff. It also does away with the need for subjective decisions to be made about which staff and outputs to select and by extension the pernicious effects of using journal ranking lists, like the Chartered Association of Business Schools, CABS (Mingers and Willmott 2013). More to the point, the overall approach is highly transparent, being based on publicly available data and easy to understand metrics.

Our current work also investigated the possibility of normalizing the data based on the mixture of subjects at each university through the use of linear regression, i.e., to account for much higher citation rates observed in the fields of science and medicine. The resulting ranking did correct for this although the results could be considered extreme.

In terms of limitations, this paper is only a preliminary investigation of the feasibility of using GS citation data for judging research quality. Further research should look at the effects of using different numbers of staff—more or less than 50; a wider, possibly international, comparison involving a larger number of universities, and other non-REF ranking systems; and a detailed investigation into the accuracy and biases of GS data itself. It might also be possible to obtain lists of staff from Universities to avoid the GS errors.

## Footnotes

1 Indeed, after the 2008 exercise, the reviewers were explicitly told to destroy any notes they may have made during the process to avoid FOI requests or litigation.

2 We are grateful to Isidro Aguillo for this information.

3 One can achieve a similar but not identical result by typing just the domain name into the GS search box and then click “user profiles”.
